# Complete Genome Sequence and Characterization of a Protein-Glutaminase Producing Strain, *Chryseobacterium proteolyticum* QSH1265

**DOI:** 10.3389/fmicb.2018.01975

**Published:** 2018-09-04

**Authors:** Ruidan Qu, Xiaoyu Zhu, Min Tian, Yingjie Liu, Wenjuan Yan, Jian Ye, Hongliang Gao, Jing Huang

**Affiliations:** School of Life Science, East China Normal University, Shanghai, China

**Keywords:** *Chryseobacterium proteolyticum*, protein-glutaminase, deamidation, third-generation sequencing, annotation

## Abstract

Recently, an enzyme named protein-glutaminase (PG) has been identified as a new type of enzyme with significant potential for deamidation of food proteins. The enzyme is shown to be expressed as a pre-pro-protein with a putative signal peptide of 21 amino acids, a pro-sequence of 114 amino acids, and a mature PG of 185 amino acids. The microbial enzyme PG specifically catalyzes deamidation of proteins without protein hydrolysis pretreatment and only reacts with glutamine residues in the side-chains of proteins or long peptides. All these attributes suggest that it has a great potential for food industrial applications. However, until recently, there have been relatively few studies of the PG-producing strains. A strain named *Chryseobacterium proteolyticum* QSH1265 which can produce PG was isolated from a soil sample collected in Songjiang, Shanghai, China. Its enzyme activity was about 0.34 ± 0.01 U/mL when using carboxybenzoxy-Gln-Gly as a substrate. The strain can produce acid from D-glucose, maltose, L-arabinose sucrose, glycerol, and mannitol but not fructose, and it is also positive for indole production and urease. Here we describe the complete genome sequence of this strain via PacBio RSII sequencing. The *C. proteolyticum* QSH1265 genome consists of a circular chromosome with total length of 4,849,803 bp without any plasmids. All of 4563 genes were predicted including 4459 genes for protein-coding and 104 RNA-relative genes with an average G+C content of 36.16%. The KEGG and COG annotation provide information for the specific function of proteins encoded in the genome, such as proteases, chromoproteins, stress proteins, antiporters, etc. A highly conserved hypothetical protein shares a promoter with the gene encoding the protein-glutaminase enzyme. The genome sequence and preliminary annotation provide valuable genetic information for further study of *C. proteolyticum*.

## Introduction

Vegetable protein-containing (especially protein from soy) foods and beverages have become popular among consumers because of their potential health benefits. Since 2003, both the soy protein-based market and sales have shown a strong increase (Suppavorasatit et al., 2013). However, vegetable proteins usually contain a high level of glutamine and asparagine which may crosslink with other amino acids through hydrogen bonds, resulting in low solubility and undesirable “off” flavors in aqueous solutions (Shih et al., 1992). Low solubility has limited the utilization of proteins, especially vegetable proteins in the food industry. Low solubility and “off” flavors can be solved by changing the conformation of proteins by physical, chemical, and enzymatic modification (Seo et al., 2008; Liu et al., 2010). Deamidation, which has proved to be one of the most promising protein modification methods, can improve the solubility, emulsification, foaming, and other functional properties of food proteins by increasing the number of negative charges that decrease the isoelectric point of the protein (Hamada and Swanson, 1994), which results from converting amide groups into carboxyl groups with the concomitant release of ammonia.

Due to its high efficiency, mild reaction conditions, strong specificity, and safety, enzymatic protein deamidation is becoming more desirable than chemical and physical treatments for food systems (Panyam and Kilara, 1996). Some enzymes, transglutaminase, peptidoglutaminases, and proteases for this purpose have been explored. However, side reactions are inevitable for transglutaminase and proteases due to the primary catalytic reactions of these two enzymes are not deamidation itself, and the substrates of peptidoglutaminases are limited to short-size peptides (Kikuchi et al., 1971). Thus, an ideal enzyme is required that catalyzes the deamidation of protein rather than short peptides.

Protein-glutaminase (PG) is a novel deamidation enzyme obtained from purified culture supernatant of *Chryseobacterium proteolyticum* strain 9670T (Yamaguchi and Yokoe, 2000). Compared with the other deamidation enzymes, deamidation of protein is the primary catalytic reaction of PG. It only reacts with glutamine residues in the side-chains of proteins or long peptides, instead of asparagine residues and free glutamine. According to previous reports, PG is a monomeric single polypeptide consisting of 185 amino acids with an isoelectric point of 10.0 and a molecular weight of 19.86 kDa. The enzyme is expressed as a pre-pro-protein with a putative signal peptide of 21 amino acids, a pro-region of 114 amino acids and a mature PG of 185 amino acids (Yamaguchi et al., 2001).

The species *C. proteolyticum*, belongs to the genus *Chryseobacterium* of the family Flavobacteriaceae, (**Table 1**) which can produce PG. The safety of both the strain and its production was verified in 2007 (Scheuplein et al., 2007). Previous studies had a much narrower focus, concentrating on the properties, structure, and applications of PG instead of the enzyme producing strain (Yamaguchi et al., 2001; Yong et al., 2004, 2006; Miwa et al., 2010; Liu et al., 2011; Cui et al., 2013; Miwa et al., 2013). It is urgent to enrich strains to improve the production of PG. A PG producing strain, *C. proteolyticum* QSH1265, was the first wild strain isolated in April, 2014 from soil in the Songjiang district of Shanghai, China by enrichment cultivation with carboxybenzoxy (CBZ)-Gln-Gly as the only nitrogen source. Its enzyme activity was about 0.34 ± 0.01 U/mL when using CBZ-Gln-Gly as a substrate (**Supplementary Figure S1**). However, the low enzyme producing ability of wild strains, as well as the genomic diversity and an incomplete understanding of the genetic features of *C. proteolyticum*, has greatly limited the application of PG in the food industry. Here, we present the genome sequence and the genomic information of *C. proteolyticum* QSH1265.

**Table 1:** Classification and general features of *C. proteolyticum* QSH1265 according to the MIGS recommendations.

MIGS	Property	Term	Evidence code
	Classification	Domain *Bacteria*	TAS
		Phylum *Bacteroidetes*	TAS
		Class	TAS
		Order	TAS
		Family *Flavobacterium*	TAS
		Genus *Chryseobacterium*	TAS
		Species *C. proteolyticum*	TAS
		Strain: *C. proteolyticum* QSH1265	TAS
	Gram stain	Gram-negative	TAS
	Cell shape	Rod	TAS
	Motility	Non-motility	TAS
	Sporulation	None	TAS
	Temperature range	Mesophile	TAS
	Optimum temperature	36–37°C	TAS
	pH range	pH 5.0–11.0	TAS
	Carbon source	Glucose, sucrose, mannitol	TAS
MIGS-6	Habitat	Soil, fresh water, fish, diary food	IDA
MIGS-22	Oxygen requirement	Aerobic	TAS
MIGS-23	soil		
MIGS-15	Biotic relationship	Free-living	IDA
MIGS-14	Pathogenicity	Safe	TAS
MIGS-4	Geographic location	Shanghai, China	This study
MIGS-5	Sample collection time	2014	This study
MIGS-4.1	Longitude	121.24	This study
MIGS-4.2	Latitude	31.0	This study
MIGS-4.3	Depth	0.1–0.5 m above the surface	This study
MIGS-4.4	Altitude	98 m	This study


*Evidence codes - IDA: Inferred from Direct Assay; TAS: Traceable Author Statement (i.e., a direct report exists in the literature).*

## Materials and Methods

### Bacterial Strain Information and Phenotypic Characteristics

**FIGURE 1:**
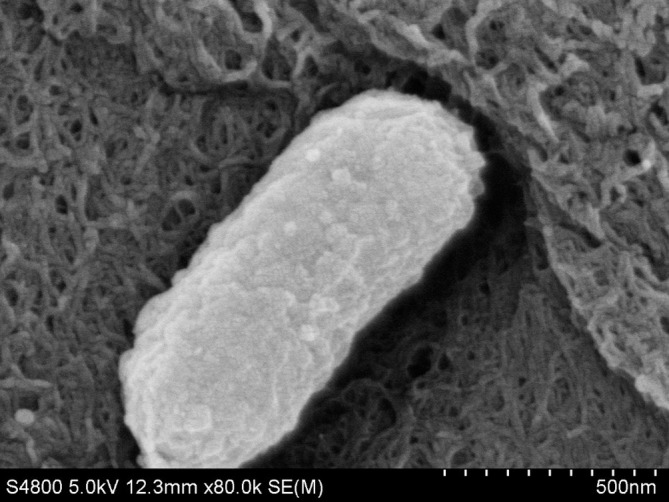
Scanning electron micrograph of strain *C. proteolyticum* QSH1265 cells from 12 h of culture. Strain QSH1265 is a medium rod-shaped bacterium with a length of 0.5 to 1.8 μm and a diameter of 0.3–0.6 μm. The scale bar, indicates 0.5 μm.

The cells of *C. proteolyticum* QSH1265 are Gram-negative, aerobic, non-spore forming bacteria. Lacking flagella, they are smooth surfaced, short and rod-shaped; in alkaline environments they turn red (Yamaguchi and Yokoe, 2000). Electron microscopy reveals that the length of cells varies from 0.5 to 1.8 μm and the width is between 0.3 and 0.6 μm (**Figure 1**). The optimum temperature and pH environment for growth for this organism is 36–37°C and pH 5 to 11 (**Table 1**). *C. proteolyticum* QSH1265 can produce acid from D-glucose, maltose, L-arabinose sucrose, glycerol, and mannitol but not fructose. It is also positive for indole production and the activity of catalase and urease but negative for hydrolysing of starch (data not shown).

### Growth Conditions and Genomic DNA Preparation

*Chryseobacterium proteolyticum* strain QSH1265 was grown in a culture medium consisting of 1.0% polypeptone, 0.2% yeast extract, and 0.1% MgSO_4_. Strains were cultured on nutrition agar plates and then inoculated in the above medium. The cells were collected after being aerobically incubated at 30°C for 12 h with reciprocal shaking at 200 r/min. The full genomic DNA of QSH1265 was extracted using the Microbial DNA extraction kit (Takara, Tokyo, Japan) according to the manufacturer’s instructions. Then 1% agarose gel electrophoresis and nanodrop spectrometer were used to detect the quality and quantity.

### Genome Sequencing and Assembly

*Chryseobacterium proteolyticum* strain QSH1265 was selected to sequence using the PacBio Rs II single Molecule Real Time (SMRT) sequencing technology, the third-generation sequencing platform at the Personalbio Co., Ltd., in Shanghai, China. The full genome sequencing and sequence assembly was completed in 2016 and its genome sequence was deposited in GenBank (SRR7156726). The strain has been preserved in the China General Microbiological Culture Collection Centre (CGMCC). Basic genome sequencing information is shown in **Table 2**.

**Table 2:** Genome sequencing project information for *C. proteolyticum* QSH1265.

MIGS	Property	Term
MIGS-31	Finishing quality	Finished
MIGS-28	Libraries used	2 PacBio SMRT cells
MIGS-29	Sequencing platforms	PacBio RS II Illumina Miseq
MIGS-30	Assembler	HGAP
MIGS-32	Genome database release GenBank ID Bioproject accession Data type	GenBank SRR7156726 PRJNA471029 Genome sequencing


All the sequencing data were assembled using the software Hierarchical Genome assembly process (HGAP), to obtain contigs. Then Mummer software was used for co-linearity analysis, which compares the relative position of each contig in the genome. Next, the gaps between contigs were filled by Illumina Miseq, the second generation sequencing platform. Finally, the sequencing results were rectified using the software, Pilon.

### Sub-Systematic Analysis and Functional Annotation of ORFs in Genome

Automated genome annotation was completed by the following ways: Gene ontology (GO) annotation was assigned to each of ORFs by Blast2GO software, which analyzed the best hits of the BLAST results (Conesa and Götz, 2008). Ortholog information and metabolic pathway annotation of protein coding genes are mainly completed by the KAAS automatic annotation system of Kyoto Encyclopedia of Genes, Genomes (KEGG) (Moriya et al., 2007). The whole genome coding DNA sequences (CDSs) and transfer RNAs were identified using the software, Glimmer 3.0 (Delcher et al., 1999) and tRNAscan-SE 1.4 (Lowe and Eddy, 1997). Ribosomal RNAs were predicted using RNAmmer1.2 (Lagesen et al., 2007). Directly repeated sequences (DRs) and spacers of the full genome sequences were predicted with the CRISPR recognition tool (Bland et al., 2007). Additionally, the gene island was predicted using IslandViewer (Langille and Brinkman, 2009), whereas signal peptide sequences and genes with transmembrane helices were predicted using the software SignalP 4.1 and TransMembrane prediction using Hidden Markov Models (TMHMM). Finally, the DNA sequence, gene and predicted non-coding RNA sequences were integrated into the standard GenBank format, then the circle map of the genome was drawn in CGView (Stothard and Wishart, 2005).

## Results

### 16S rRNA Gene Sequence and Analysis

**FIGURE 2:**
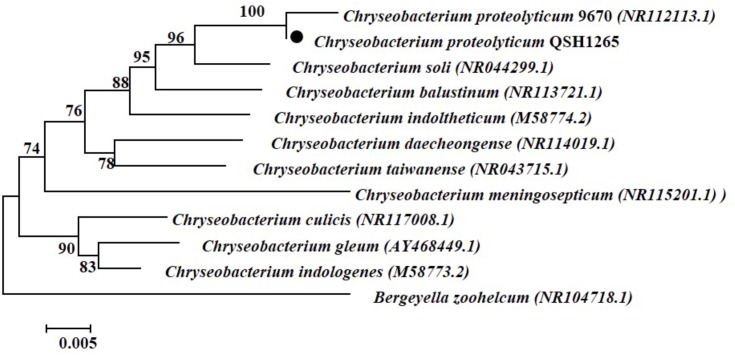
Phylogenetic tree showing the position of *C. proteolyticum* QSH1265 (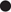), the type strains of other species in the genus *Chryseobacterium*, and the type strain of *Bergeyella zoohelcum*. The sequences were aligned with the CLUSTAL W program and were constructed with the neighbor-joining algorithm integrated in the MEGA 7.0 program. The phylogenetic tree was tested with 1000 bootstrap replicates. Bootstrap values are shown at the nodes. The GenBank accession numbers of the sequences are indicated the parentheses. The scale bar represents a 0.5% nucleotide sequence divergence.

The 16S rRNA gene sequences of QSH1265 were compared with others from NCBI database using the Basic Local Alignment Search Tool (BLAST). After aligning the sequences using the Clustal X (v1.81) program, the phylogenetic tree was constructed with the neighbor-joining algorithm integrated in the MEGA 7.0 program. This showed that the clustering of strain QSH1265 was consistent with other species of the genus *Chryseobacterium*. Analysis of the 16S rRNA sequence revealed that QSH1265 is most closely related to *C. proteolyticum* strain 9670 (NR 112113.1) and it shares 99% homology and are grouped in one branch of the genus *Chryseobacterium* (**Figure 2**). QSH1265 was shown to be a distinct branch, but sharing 94% homology with the other strains, which also belong to the genus *Chryseobacterium*, such as *Chryseobacterium meningosepticum* (NR 115201.1), *Chryseobacterium taiwanense* (NR 043715.1). QSH1265 was clearly distant from *Bergeyella zoohelcum* (NR104718.1), a phylogenetically related species that is also a member of the Flavobacteriaceae family.

### Genome Properties

**FIGURE 3:**
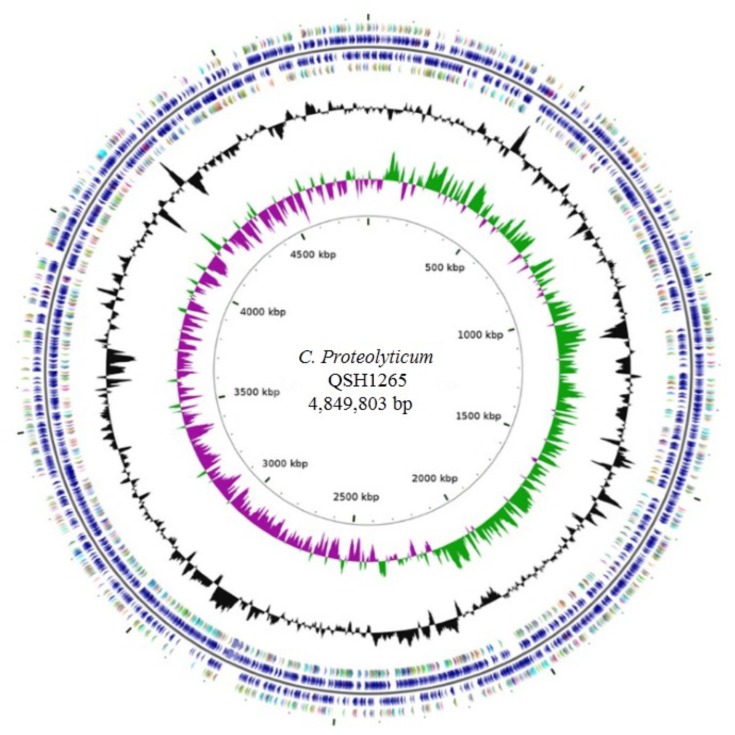
Graphical circular map of the chromosome of *C. proteolyticum* QSH1265. From center to the outside: the scales in the first circle indicate location in Mbp, strating with the initial coding region. The second circle shows the GC skew (in purple and green), and the value is plotted as the deviation from the average GC skew of the entire chromosome sequence. The bar in the third circle (in black and red) represents the GC content. The bars in the forth and seventh circle are colored according to COG function categories of CDS. Position of tRNA and rRNA are marked by brown and purple in the fifth and sixth circle, respectively.

**FIGURE 4:**
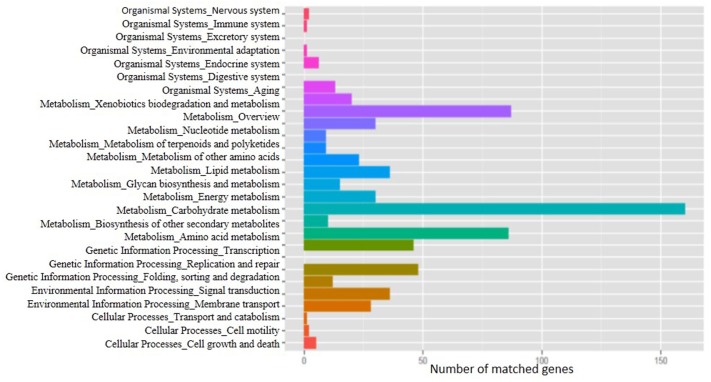
KEGG classification of *C. proteolyticum* QSH1265. A total of 872 genes were predicted using the KEGG pathway annotation. On the left is listed the classification of the functional genes. Different colors on the right side represent different metabolic pathways.

The whole genome of *C. proteolyticum* strain QSH1265 contains a single chromosome of 4,849,803 bp (**Figure 3**) and has an average G+C content of 36.16%. In total, 4563 genes were identified including 4459 protein-coding genes; in addition, 104 genes for RNA species-including 15 rRNA genes, 69 tRNA genes, and 20 ncRNA genes have been determined. The genome properties of QSH1265 are shown in **Table 3**. GO, KEGG and the clusters of orthologous genes (COG) annotation indicate the information of specific functional proteins. COG functional categories are listed in **Table 4**, in which 2675 of the identified genes were classified: 145 genes for translation, ribosomal structure and biogenesis, 221 genes for transcription, 121 genes for replication, recombination and repair, 16 genes for cell cycle control, cell division and chromosome partitioning, 58 genes for defense mechanisms, 127 genes for signal transduction mechanisms, and 851 genes for metabolism and so on. In addition, GO and KEGG functional categories are shown in **Figure 4**. Genome analysis revealed that *C. proteolyticum* strain QSH1265 had many functions although its genome size was relatively small. According to the analysis and forecast results of these genome, 4459 ORFs, 906 transmembrane helices, 489 signal peptides, 110 genomic islands were predicted.

**Table 3:** Nucleotide content and gene count levels of genome.

Attribute	Value	% of total
Size (bp)	4,849,803	100.00
G+C content (bp)	1,753,689	36.16
Coding regions (bp)	4,324,467	89.17
Total genes	4,563	100.00
RNA genes	104	2.28
Protein-coding genes	4,459	97.72
Genes assigned to COGs	3,759	82.38
Genes with signal peptides	489	10.97
Genes with transmembrane helices	906	20.32


**Table 4:** Number of genes associated with the 25 general COG functional categories.

Code	Value	% of total	Description
J	145	3.25	Translation, ribosomal structure, and biogenesis
A	0	0.00	RNA processing and dynamics
K	221	4.96	Transcription
L	121	2.71	Replication, recombination, and repair
B	0	0.00	Chromatin structure and dynamics
D	16	0.36	Cell cycle control, cell division, chromosome partitioning
Y	0	0.00	Nuclear structure
V	58	1.30	Defense mechanisms
T	127	2.85	Signal transduction mechanisms
M	178	3.99	Cell wall/membrane/envelope biogenesis
N	5	0.11	Cell motility
Z	0	0.00	Cytoskeleton
W	0	0.00	Extracellular structure
U	33	0.74	Intracellular trafficking, secretion, and vesicular transport
O	94	2.11	Posttranslational modification, protein turnover, chaperones
C	153	3.43	Energy production and conversion
G	175	3.92	Carbohydrate transport and metabolism
E	235	5.27	Amino acid transport and metabolism
F	57	1.28	Nucleotide transport and metabolism
H	114	2.56	Coenzyme transport and metabolism
I	118	2.65	Lipid transport and metabolism
P	152	3.41	Inorganic ion transport and metabolism
Q	92	2.06	Secondary metabolites biosynthesis, transport, and catabolism
R	399	8.95	General function prediction only
S	182	4.08	Function unknown
–	1784	40.01	Not in COGs


### Analysis of Hypothetical Protein

A hypothetical protein located in the ORF of 3589 was predicted to be a highly conserved protein using the online analysis software program ProParam^1^. Analysis using the Softberry software^2^ suggested that it might share a promoter with the gene encoding the PG (the sequence was showed in **Supplementary Figure S2**). Analysis using SignalP 4.1^3^ indicated that PG is likely expressed as a pre-pro-protein with a putative signal peptide of 22 amino acids, and a mature protein of 80 amino acids. The analysis of the signal peptide sequence through Protein Data Bank (PDB) database revealed that it is similar to anionic transporters and ATP-binding cassette (ABC) transporter substrate binding protein having respective homologies of 85 and 65%. ABC transporters are a group of integral membrane proteins which assist in transmembrane transport of various molecules (Snider et al., 2013). The result hints that this hypothetical protein may be associated with the secretory pathway of PG.

### Methylation Modification Analysis

It is widely known that methylation modification is closely associated with gene transcription activity and significantly affects gene expression. Genome methylation modification and methyl-transferase recognition sequence motifs were analyzed using SMRT 2.3.0. In the chromosome, 322034 ^m4^C (N4-methylcytosine), 17830 ^m6^A (N6-methyladenine), and 1631972 other modified bases were marked as modified. Methylation analysis also demonstrates the methylation distribution of the individual genes or intergenic regions. Corresponding annotation information is included in **Table 5**.

**Table 5:** Sequence structure and general information of motifs in the whole genome.

Motif string	CenterPos	Type	Fraction	nDetectd	nGenome	MeanScore	meanCoverage
GGANNNNNTCG	2	m6A	0.9815	902	919	212.969	178.49
CGANNNNNTCC	2	m6A	0.9793	900	919	208.0344	179.42


## Discussion

As mentioned above, PG is thought to be one of the most promising protein-deamidation enzymes and has been used to modify many proteins, including wheat gluten (Yong et al., 2006), α-Lactalbumin (Gu et al., 2001), α-zein (Yong et al., 2004), rice glutelin (Liu et al., 2011), skim milk (Miwa et al., 2010), soy protein (Suppavorasatit et al., 2011), yogurt (Miwa et al., 2014), and oat protein (Jiang et al., 2015). Miwa et al. (2013) investigated the effect of PG on heat-induced conformational changes in whey protein isolate and its relation to gel properties. Other researchers have focused on the protein structure changing, solubility, and flavor improvements induced and modified by PG (Cui et al., 2013; Suppavorasatit et al., 2013; Kunarayakul et al., 2017; Chen et al., 2018).

However, few studies about PG producing strains have been reported. Only characterization of a type strain *C. proteolyticum* 9670 has been published, which was isolated from soil collected in Japan by Yamaguchi and Yokoe (2000) in this strain’s enzyme producing ability was very poor, just up to 0.258 U/mL when using CBZ-Gln-Gly as a substrate. So far there’s no study reported the genome sequence of the strains.

In this study, we have reported, for the first time, the complete whole genome sequence of PG producing strain *C. proteolyticum* QSH1265, a soil bacterium isolated from Shanghai, China. The genome sequence of *C. proteolyticum* QSH1265 contains a single chromosome of 4,849,803 bp and has an average G+C content of 36.16%. In total, 4563 genes were identified including 4,459 protein-coding genes and 104 genes for RNA species. There is no plasmid in the genome, suggesting that it could be difficult to establish plasmid-based expression system. However, mapping the whole genome sequence provides valuable genetic information that can be exploited in directional mutation and transposon mutants library establishment (Chiang and Rubin, 2002). PG gene and its related genes were also detected. On the basis of this information, we could further explore some genetic tools to obtain high yield strains and reveal the bio-function of PG. Further research into resequencing (Li et al., 2009) and RNA-seq (Wang et al., 2009) of high yield strains is required to help the long-term study of *C. proteolyticum* and develop its potential use in the food industry.

## Author Contributions

RQ and XZ designed and performed experiments, acquired data, wrote, revised, and approved final manuscript. These authors have contributed equally to this work. MT acquired data, drafted manuscript, and approved final manuscript. YL and JY interpreted data and approved final manuscript. WY acquired data, provide the material, and approved final manuscript. HG revised and approved final manuscript. JH conceived and supervised the project, secured funding, and revised and submitted the manuscript.

## Conflict of Interest Statement

The authors declare that the research was conducted in the absence of any commercial or financial relationships that could be construed as a potential conflict of interest.

## Footnotes

1 http://web.expasy.org/protparam/

2 http://www.softberry.com/

3 http://www.cbs.dtu.dk/services/SignalP/
