# Supplementary material for: Complete Genome Sequence and Characterization of a Protein-Glutaminase Producing Strain, Chryseobacterium proteolyticum QSH1265
Source: Front Microbiol. 2018 Sep 4;9:1975. doi: 10.3389/fmicb.2018.01975 (PMC6132073; doi:10.3389/fmicb.2018.01975)
Supplement: Supplementary file 1 [file Data_Sheet_1.docx]

Supplementary Material

**Complete genome sequence and characterization of a protein-glutaminase producing strain, *Chryseobacterium proteolyticum* QSH1265**

**Ruidan Qu^1 †^, Xiaoyu Zhu^1†^, Min Tian^1^, Yingjie Liu^1^, Wenjuan Yan^1^, Jian Ye^1^, Hongliang Gao^1^, Jing Huang^1*^**

^1^School of Life Science, East China Normal University, Shanghai, China

^†^These authors have contributed equally to this work.

*** Correspondence:**Jing Huang

[jhuang@bio.ecnu.edu.cn](mailto:jhuang@bio.ecnu.edu.cn)

**Supplementary Figure 1:**


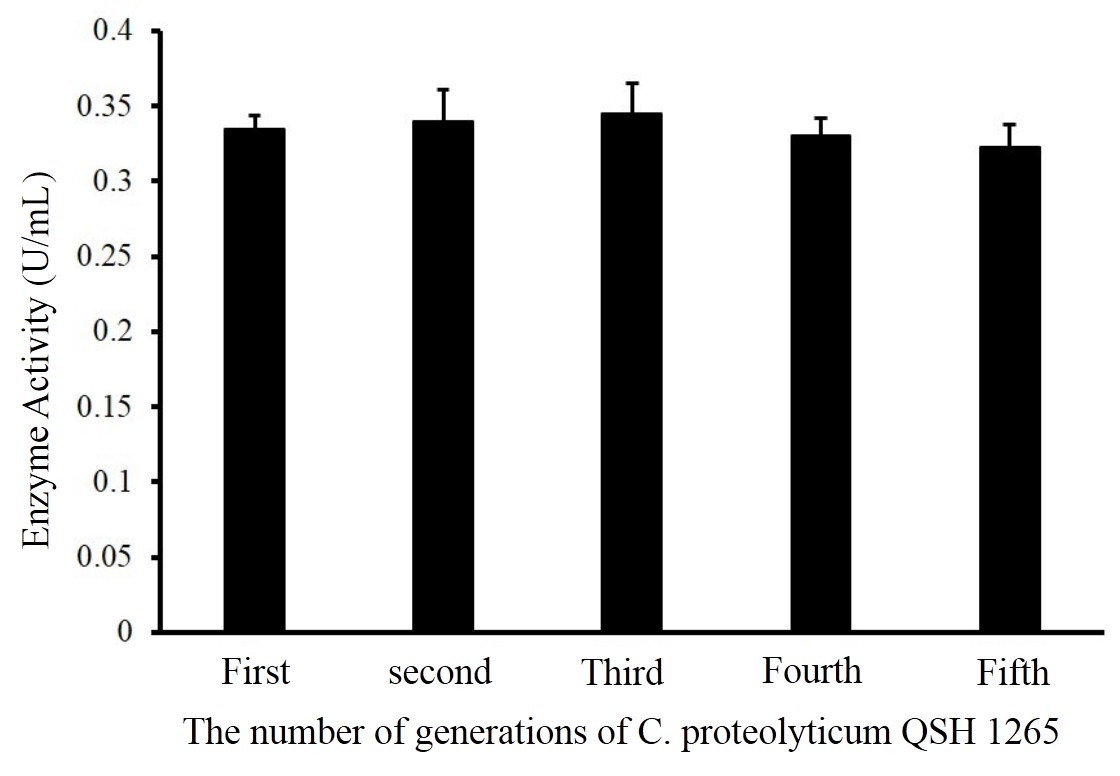


The number of generations of *C.proteolyticum* QSH1265.

01

04

10

07

15

**Fig.S1** Genetic stability of *Chryseobacterium proteolyticum* QSH1265. It took five weeks to verify the stability of PG producing strain QSH1265 and during the observed time the strain was shown to maintain stable PG activity for at least 15 generations. The results showed that the average enzyme activity of 01, 04, 07, 10 and 15 generations was about 0.34±0.01U/mL when using carboxybenzoxy-Gln-Gly as a substrate. Data represent mean ± SD; n = 3 independent experiments.

**Supplementary Figure 2:**

**
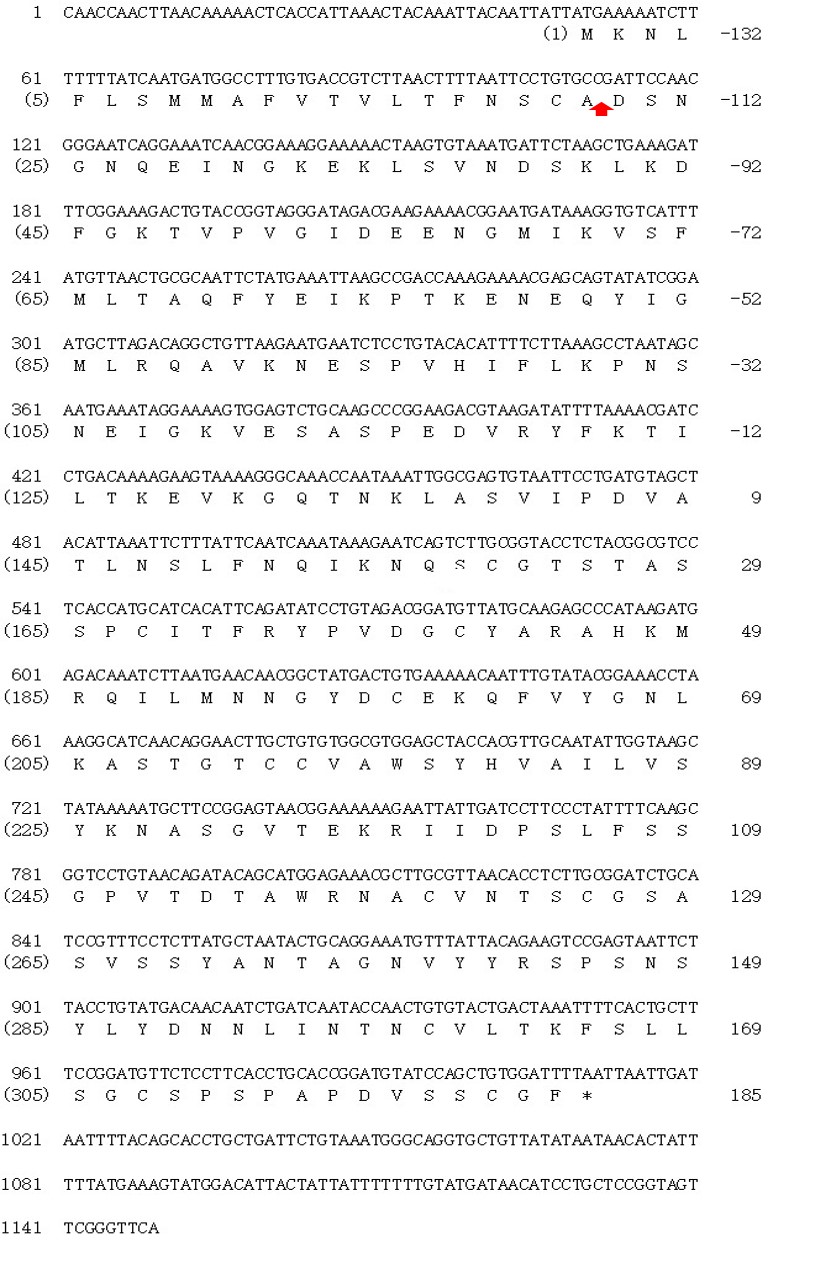
**

**Fig.S2** Nucleotide sequence of the protein-glutaminase gene and its deduced amino-acid sequence. The red vertical arrow indicates the putative signal peptide cleavage site. Nucleotide numbering is indicated on the left. The amino-acid numberings start at the translation initiation codon (in parentheses) and the N-terminus of the mature enzyme (on the right) at +1.
